# Association of parental HLA-G polymorphisms with soluble HLA-G expressions and their roles on recurrent implantation failure: A systematic review and meta-analysis

**DOI:** 10.3389/fimmu.2022.988370

**Published:** 2022-12-01

**Authors:** Lian Hu, Dongmei He, Hong Zeng

**Affiliations:** ^1^ Department of Gynecology and Obstetrics, The Fourth Changsha Hospital, Changsha, China; ^2^ Department of Gynecology and Obstetrics, Sun Yat-Sen Memorial Hospital, Sun Yat-Sen University, Guangzhou, China; ^3^ Department of Reproductive Medicine Center, Foshan Maternal and Child Health Care Hospital, Southern Medical University, Guangzhou, China; ^4^ Department of Reproductive Medicine Center, Xiangya Hospital, Central South University, Changsha, China

**Keywords:** Human leukocyte antigen G, immune tolerance, recurrent implantation failure, genetic polymorphism, meta-analysis

## Abstract

**Introduction:**

HLA-G plays a central role in immune tolerance at the maternal-fetal interface. The HLA-G gene is characterized by low allelic polymorphism and restricted tissue expression compared with classical HLA genes. HLA-G polymorphism is associated with HLA-G expression and linked to pregnancy complications. However, the association of parental HLA-G polymorphisms with soluble HLA-G (sHLA-G) expression and their roles in recurrent implantation failure (RIF) is unclear. The study aims to systematically review the association of HLA-G polymorphisms with RIF, the association of sHLA-G expression with RIF, and the association of HLA-G polymorphisms with sHLA-G expressions in patients attending *in-vitro* fertilization (IVF) treatment.

**Methods:**

Studies that evaluated the association of HLA-G polymorphisms with RIF, the association between sHLA-G expression with RIF, and the association between HLA-G polymorphisms with sHLA-G expressions in patients attending IVF treatment were included. Meta-analysis was performed by random-effect models. Sensitivity analysis was performed by excluding one study each time. Subgroup analysis was performed based on ethnicity.

**Results:**

HLA-G 14bp ins variant is associated with a lower expression of sHLA-G in seminal or blood plasma of couples attending IVF treatment. The maternal HLA-G*010101 and paternal HLA-G*010102 alleles are associated with RIF risk compared to other alleles. However, single maternal HLA-G 14bp ins/del polymorphism, HLA-G -725 C>G/T polymorphism, or circulating sHLA-G concentration was not significantly associated with RIF in the general population. HLA-G 14bp ins/ins homozygous genotype or ins variant was associated with a higher risk of RIF in the Caucasian population.

**Discussion:**

Specific HLA-G alleles or HLA-G polymorphisms are associated with sHLA-G expression in couples attending IVF treatment. Several HLA-G polymorphisms may be related to RIF, considering different ethnic backgrounds. A combined genetic effect should be considered in future studies to confirm the association of HLA-G polymorphisms and sHLA-G expressions in relation to RIF.

## Introduction

Human leukocyte antigen (HLA)-G is a non-classical HLA class Ib molecule that plays a vital role in the maternal acceptance of the semi-allogenic fetus. The HLA-G gene, locates on chromosome 6p21.3, consists of seven introns and eight exons. Alternative splicing of HLA-G mRNA generates seven HLA-G isoforms, including four membrane isoforms (G1-G4) and three soluble isoforms (G5-G7) (1). In addition, soluble HLA-G (sHLA-G) can be generated by shedding or proteolytic cleavage of membrane-anchored HLA-G through matrix metalloproteinases (MMPs) activity, such as shed HLA-G1 (2, 3). HLA-G gene is characterized by low allelic polymorphism and restricted tissue expression compared with highly polymorphic classical HLA Ia genes (HLA-A, B, C). HLA-G’s expression is mainly restricted to the maternal-fetal interface and immune tissues. HLA-G is detectable in body fluids as secreted soluble molecules despite the restricted tissue expression (4–6). Essential functions of HLA-G at the fetal-maternal interface include the inhibition of natural killer (NK) cells mediated cytolysis, enrichment of regulatory T (Treg) cells, and promotion of a shift from a T-helper (Th)1 to a Th2 cytokine profile (7). HLA-G polymorphisms are associated with abnormal HLA-G levels and linked to reproductive disorders such as implantation failure, recurrent miscarriage, preeclampsia, and placental abruption (8–14). One of the most studied HLA-G polymorphisms is the 14bp insertion/deletion (ins/del) polymorphism located on exon eight at the 3’ untranslated region (3’UTR). HLA-G 14bp ins/del affects the stability of HLA-G mRNA and leads to abnormal HLA-G expression (15, 16), which is associated with recurrent miscarriage (11). HLA-G -725C>G polymorphism located at the 5’upstream regulator region (5’URR) or promoter region is reported to change the methylation profile of CpG dinucleotide, resulting in a modification of HLA-G expression and also linked to miscarriage (17). Besides, the other HLA-G polymorphism such as HLA-G -964G>A at 5’ URR, HLA-G allele variation at exon 2, 3, 4, intron 2, and specific HLA-G haplotypes/diplotypes are associated with sHLA-G expression and may be linked to reproductive outcomes (8, 18–21). However, the role of HLA-G polymorphism on RIF has been investigated in only a few studies with contradictory results.

Recurrent implantation failure (RIF) is a complication following *in-vitro* fertilization and embryo transfer (IVF-ET), with an incidence rate of approximately 10~15%. RIF is defined as good-quality embryos repeatedly failing to implant. It is generally diagnosed based on the number of unsuccessful ET cycles, the number of transferred embryos, female age, or a combination of these factors (22). The causes of RIF include decreased quality of gametes or embryos, decreased endometrial receptivity, uterine anomalies, immune diseases, thrombophilia conditions, endocrine disorders, metabolic disorders, and genetic abnormalities (22). Genetic factors contribute to RIF susceptibility as several genetic polymorphisms have been reported to be associated with RIF (23–26). Investigating the role of genetic polymorphisms on RIF susceptibility can help to promote our understanding of the pathogenesis underlying RIF and contributes to the prediction and prevention of RIF. Increasing evidence underlines the essential role of immune factors on embryo implantation as pregnancy remains an immune challenge for the uterus. The key to successful implantation and pregnancy maintenance is the immune tolerance of the uterus to the semi-allogeneic fetus (27). HLA-G plays a central role in immune tolerance at the maternal-fetal interface. Interactions between sHLA-G and uterine lymphocytes induce maternal immune tolerance for the invading extravillous trophoblasts, which is the critical factor affecting embryo implantation. Soluble HLA-G is essential for embryo implantation. The embryo-secreted sHLA-G in the culture medium served as a promising predictor for a successful pregnancy (28–32). However, the role of parental sHLA-G expression before pregnancy is less studied. The sHLA-G expression in circulating blood is significantly increased in pregnant women compared to that of unpregnant women. sHLA-G level is dynamically changed during pregnancy. Recent studies indicate that HLA-G may be involved in preparing for an immune environment before embryo implantation because sHLA-G can be detected in the genital tract, endometrium, and circulating blood of unpregnant women and is also present in male semen. Though maternal sHLA-G levels before pregnancy have been measured, their relationship with RIF has not yet been well established. Therefore, the current study aims to investigate the association of HLA-G polymorphisms with RIF, the association of sHLA-G expression with RIF, and the association of HLA-G polymorphisms with sHLA-G expression in patients attending IVF treatment.

## Materials and methods

The authors performed this meta-analysis following the Preferred Reporting Items for Systematic Reviews and Meta-Analysis (PRISMA) guideline (33).

### Searching strategy

We searched EMBASE, Pubmed, and CNKI (China National Knowledge Infrastructure) for related studies from their inception to 19 September 2022. The grey literature was searched in OpenGrey (http://www.opengrey.eu/). The references of the included studies were also hand-searched. For searching studies that evaluate the association between HLA-G polymorphism with RIF, the searching syntax in PubMed involves the following text words: “HLA-G” or “HLAG” or “human leukocyte antigen G” in combination with “polymorphism” or “mutation” or “allele” or “genotype” or “genetic” or “variant” or “haplotype” or “diplotype” and in combination with “implantation” or “*in vitro* fertilization” or “IVF” or “ICSI” or “Intracytoplasmic sperm injection” or “embryo transfer”. For searching studies that evaluate the association between sHLA-G expression with RIF, the searching syntax in Pubmed involves the following text words: “HLA-G” or “HLAG” or “human leukocyte antigen G” or “sHLA-G” or “sHLAG” in combination with “expression” or “level” or “concentration” and in combination with “implantation” or “*in vitro* fertilization” or “IVF” or “ICSI” or “Intracytoplasmic sperm injection” or “embryo transfer”. For searching studies that evaluate the association between HLA-G polymorphism with sHLA-G expression in patients attending IVF treatment, the searching syntax in Pubmed involves the following text words: “HLA-G” or “HLAG” or “human leukocyte antigen G” or “sHLA-G” or “sHLAG” in combination with “expression” or “level” or “concentration” and in combination with “implantation” or “*in vitro* fertilization” or “IVF” or “ICSI” or “Intracytoplasmic sperm injection” or “embryo transfer” in combination with “polymorphism” or “mutation” or “allele” or “genotype” or “genetic” or “variant” or “haplotype” or “diplotype” The detailed searching strategies and searching results in Pubmed, Embase, and CNKI were listed in the **Supplementary Material**.

### Inclusion criteria and exclusion criteria

For evaluating the association between HLA-G polymorphism with RIF or the association between sHLA-G expression with RIF, the inclusion criteria were: (1) case-control studies; (2) the cases were RIF patients; (3) the control patients were fertile women with ≥1 normal pregnancy and lived birth or infertile women with ≥1 normal pregnancy following IVF; (4) genotype frequencies or HLA-G concentrations are eligible for calculation. The exclusion criteria were: (1) non-case-control studies (cohort studies, reviews, case reports, or meta-analyses); (2) the case group was not RIF patients. For evaluating the association between HLA-G polymorphism with sHLA-G expression, the inclusion criteria were: (1) case-control studies or cohort studies; (2) the study population was patients attending IVF treatment; (3) sHLA-G expression was compared in each HLA-G genetic group. The exclusion criteria were: (1) non-case-control or non-cohort studies (reviews, case reports, or meta-analysis); (2) the study population was not patients attending IVF treatment; (3) patients with pregnancy complications (recurrent miscarriage, pre-eclampsia) or other immune diseases. Studies in all languages were included. Conference literature was included if the data was eligible for analysis and did not overlap with the published papers. Two authors (Lian Hu and Dongmei He) independently performed the study selection. A meta-analysis was performed for each HLA-G polymorphism with two or more published studies.

### Data collection and quality assessment

Two authors (Lian Hu and Dongmei He) independently extracted the data from each study. We extracted the following information from studies that evaluate associations between HLA-G polymorphism or sHLA-G expression with RIF: first author, publication year, country, ethnicity, age of cases and controls, number of cases and controls, sample origin, genotyping method, and the definition of RIF and control. We extracted the following information from studies that evaluate the association between HLA-G polymorphism with sHLA-G expression in patients attending IVF treatment: first author, publication year, country, ethnicity, study population, number of patients tested, the sample tested, the timing of sample collection, type of assay, sHLA-G isoform, detection limit, and result. We evaluated study quality following the modified Newcastle-Ottawa scale (NOS). The scores of NOS ranged from 0 points (worst) to 9 points (best). We defined scores ≥ 7 as high quality. All the other scores indicate low quality. Two authors (Lian Hu and Dongmei He) assessed the study quality independently. Disagreement in study selection, data extraction, and quality assessment was dependent on the third author (Hong Zeng).

### Statistical analysis

We performed the meta-analysis using the random-effects model due to each study’s heterogenous definition of RIF. The odds ratios (ORs) with 95% confidence intervals (CIs) or standardized difference in means (SMD) with 95% CIs were reported to evaluate the associations. The heterogeneity of the included studies was analyzed using the Q test and quantified using the I^2^ test. I^2^<25%, I^2^= 25-50%, I^2^= 50-75%, and I^2^>75% indicated no, moderate, large, and extreme heterogeneity, respectively. We examined the Hardy-Weinberg equilibrium (HWE) in the control group by the “GWASExactHW” R package (https://CRAN.R-project.org/package=GWASExactHW). Deviation from HWE was confirmed if the p-value<0.05. We assessed the publication bias using Begg’s test and Egger’s test. Publication bias was confirmed if the p-value of Begg’s or Eggers’ test was < 0.05. We performed subgroup analysis based on ethnicity. We performed sensitivity analysis by excluding one study each time. Though we only reported the result of the random-effect model in the manuscript, both the fixed-effects model and the random-effect model were performed in each meta-analysis with the results listed in the **Supplementary Materials**. All statistical analyses were performed using the R software (The R Foundation for Statistical Computing, version 4.1.1, https://www.r-project.org). A p-value < 0.05 was considered statistically significant.

## Results

### Study characteristics

**Figure 1:**
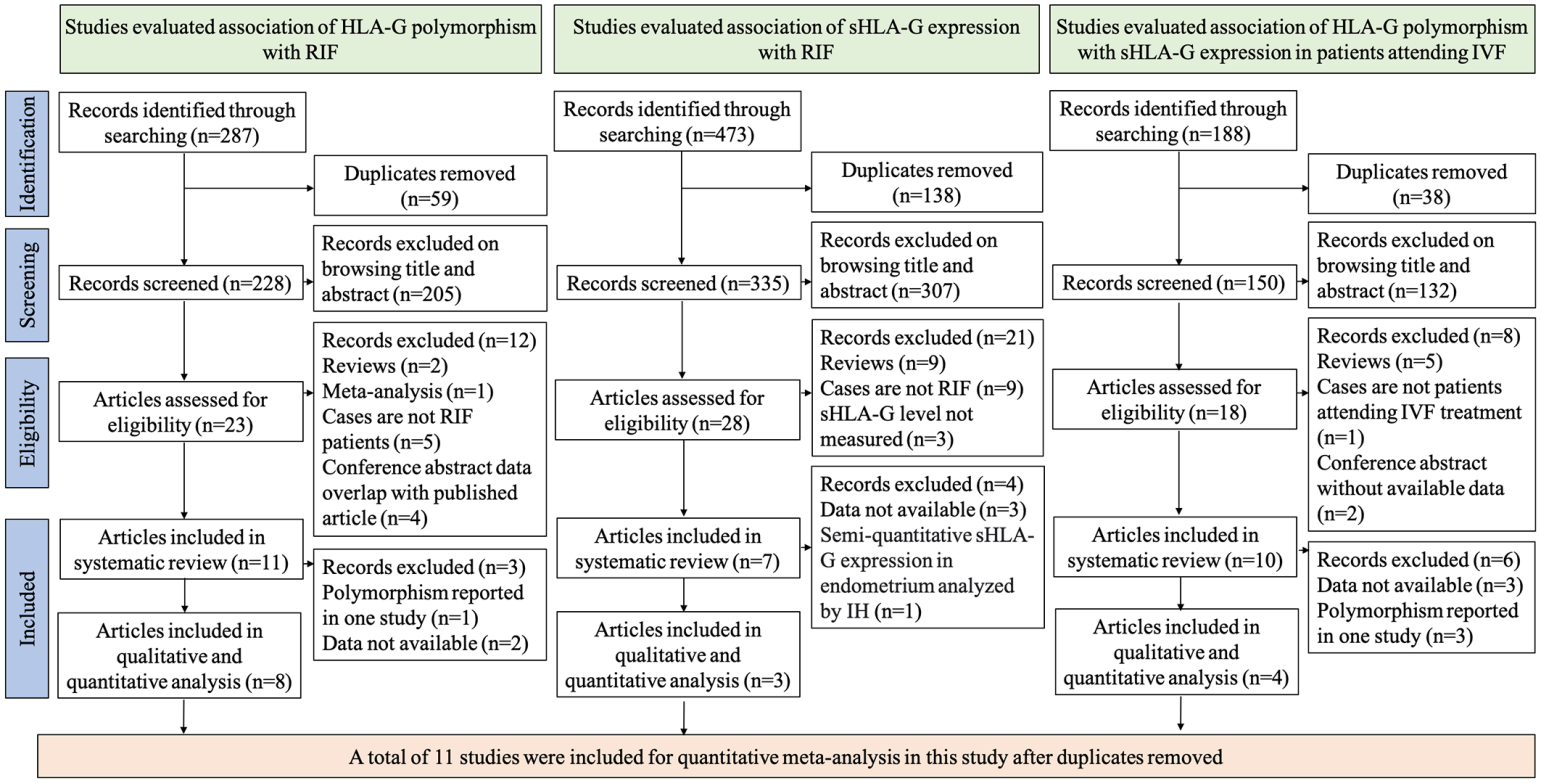
Flow chart of study selection.

To investigate the association of HLA-G polymorphism with RIF, 287 records were identified through literature searching. We excluded 59 duplicates and excluded 205 records after browsing titles or abstracts. 23 articles were assessed for eligibility (8, 9, 18–21, 34–50). 12 articles were excluded from systematic review and meta-analysis [two are reviews (38, 40), one is a meta-analysis (9), five cases are not RIF patients (18, 21, 34, 48, 49), and four are conference abstracts whose data overlap with published articles (44–47)]. 11 articles were included for systematic review. However, three articles were excluded from the quantitative meta-analysis for reasons [two articles’ data are not available to extract for synthesis (41, 43), one study reported the polymorphisms only in their research thus could not be synthesized (36)]. Finally, eight studies were included in the quantitative analysis (8, 19, 20, 35, 37, 39, 42, 50). To investigate the association of sHLA-G expression with RIF, 473 records were identified through literature searching. We excluded 138 duplicates and excluded 307 records after browsing titles or abstracts. 28 articles were assessed for eligibility (8, 19, 35, 38, 39, 41, 42, 50–70). 21 articles were excluded from systematic review and meta-analysis [nine are reviews (38, 51–58), nine cases are not RIF patients (59–67), and three studies did not measure the sHLA-G levels (39, 42, 50)]. Four studies are excluded from quantitative analysis for reasons [three articles’ data are not available to extract for synthesis (8, 68, 69), one study measured sHLA-G levels in the endometrium by semi-quantitative method (41)]. Three articles were included in the quantitative synthesis (19, 35, 70). To investigate the association of HLA-G polymorphism with sHLA-G expression in patients attending IVF treatment, 188 records were identified through literature searching. We excluded 38 duplicates and excluded 132 records after browsing titles or abstracts. 18 articles were assessed for eligibility. Eight articles were excluded from the systematic review [five were reviews (57, 58, 71–73), two were conference abstracts without data (74), and cases in one study were not IVF patients (59)]. Six studies were excluded from the quantitative meta-analysis [three studies’ data are not available to extract for synthesis (19, 41, 75), three articles reported the polymorphism in only one study thus cannot be synthesized (8, 70, 76)]. Finally, four studies were included in the quantitative meta-analysis (35, 77–79). The flow diagram of selecting studies for systematic review and meta-analysis is shown in **Figure 1**.

There are several HLA-G polymorphisms reported in different studies. Meta-analysis is performed if at least two studies evaluate the same HLA-G polymorphism with RIF, and the data can be extracted. **Table 1** summarizes the different HLA-G polymorphisms that have been reported in RIF. Association of HLA-G 14bp ins/del polymorphism, HLA-G -725 C>G polymorphism, HLA-G alleles distribution at exon2-4 (HLAG*010101, HLAG*010102, HLAG*010103, HLAG*010106, HLAG*010107, HLAG*010108, HLAG*010401, HLAG*010403, HLAG*010404, HLAG*0106, HLAG*0105N) were reported in more than two studies; therefore, meta-analyses were performed in these HLA-G polymorphisms. Meta-analysis is also performed based on three studies that evaluated the association between sHLA-G levels and RIF. The characteristics of included studies in the meta-analysis that evaluate the association between HLA-G polymorphism with RIF or the association between sHLA-G expression with RIF are presented in **Table 2**. The studies were conducted in Poland, Denmark, Brazil, Iran, and the Netherlands. The HLA-G 14bp ins/del genotype frequency in the control group has deviated from HWE in Hviid’s study (p-value=0.042) (39). The other studies’ HLA-G 14bp ins/del genotype frequencies did not deviate from HWE. The HLA-G -725C>G genotype frequencies in the included studies did not deviate from HWE.

**Table 1:** Summary of the HLA-G polymorphisms that have been reported in RIF patients.

HLA-G polymorphisms	Location	No. studies	Publication	Included in meta-analysis
**HLA-G genotypes**				
HLA-G 14-bp ins/del (rs66554220/rs371194629)	3′UTR	6	Hviid 2004 (39) Sipak-Szmigie 2009 (42) Enghelabifar 2014 (37) Lashley 2014 (50) Nardi 2016 (35) Nowak 2019 (8)	Yes
HLA-G -725 C>G(T) (rs1233334)	5′URR	2	Sipak-Szmigie 2009 (42) Nowak 2019 (8)	Yes
HLA-G -964G>A (rs1632947)	5′URR	1	Nowak 2019	No
**HLA-G haplotypes**				
Haplotypes of rs1632947-rs1233334-rs371194629	–	1	Nowak 2019 (8)	No
Haplotypes of HLA-G alleles and rs371194629	–	1	Nardi 2012b (36)	No
**HLA-G diplotypes**				
Diplotypes of rs1632947–rs1233334–rs371194629	–	1	Nowak 2019 (8)	No
**HLA-G allele distribution**				
HLA-G*010101	Exon2-4	3	Kuroshli 2015 (20) Nardi 2012a (19) Sipak-Szmigie 2009 (42)	Yes
HLA-G*01010106	Exon2-4	1	Kuroshli 2015 (20)	No
HLA-G*010102	Exon2-4	3	Kuroshli 2015 (20) Nardi 2012a (19) Sipak-Szmigie 2009 (42)	Yes
HLA-G*010103	Exon2-4	3	Kuroshli 2015 (20) Nardi 2012a (19) Sipak-Szmigie 2009 (42)	Yes
HLA-G*010105	Exon2-4	1	Kuroshli 2015 (20)	No
HLA-G*010106	Exon2-4	3	Kuroshli 2015 (20) Nardi 2012a (19) Sipak-Szmigie 2009 (42)	Yes
HLA-G*010107	Exon2-4	2	Kuroshli 2015 (20) Nardi 2012a (19)	Yes
HLA-G*010108	Exon2-4	3	Kuroshli 2015 Nardi 2012a (19) Sipak-Szmigie 2009 (42)	Yes
HLA-G*010109	Exon2-4	1	Sipak-Szmigie 2009 (42)	No
HLA-G*010112	Exon2-4	1	Nardi 2012a (19)	No
HLA-G*010114	Exon2-4	1	Nardi 2012a (19)	No
HLA-G*010120	Exon2-4	1	Nardi 2012a (19)	No
HLA-G*0103	Exon2-4	1	Kuroshli 2015 (19)	No
HLA-G*010301	Exon2-4	1	Nardi 2012a (19)	No
HLA-G*010401	Exon2-4	3	Kuroshli 2015 (20) Nardi 2012a (19) Sipak-Szmigie 2009 (42)	Yes
HLA-G*010403	Exon2-4	2	Kuroshli 2015 (20) Nardi 2012a (19)	Yes
HLA-G*010404	Exon2-4	2	Kuroshli 2015 Nardi 2012a (19)	Yes
HLA-G*0106	Exon2-4	3	Kuroshli 2015 (20) Nardi 2012a (19) Sipak-Szmigie 2009 (42)	Yes
HLA-G*01:05N	Exon2-4	3	Kuroshli 2015 (20) Nardi 2012a (19) Sipak-Szmigie 2009 (42)	Yes
HLA-G +482T/C	Intron2	1	Kuroshli 2015 (20)	No
HLA-G +485G/T	Intron2	1	Kuroshli 2015 (20)	No
HLA-G +494A/C	Intron2	1	Kuroshli 2015 (20)	No
HLA-G +505/+506 -/CC	Intron2	1	Kuroshli 2015 (20)	No
HLA-G +506 -/C	Intron2	1	Kuroshli 2015 (20)	No
HLA-G +615 A/-	Intron2	1	Kuroshli 2015 (20)	No
HLA-G +636C/T	Intron2	1	Kuroshli 2015 (20)	No
HLA-G +644G/T	Intron2	1	Kuroshli 2015 (20)	No
HLA-G +685G/A	Intron2	1	Kuroshli 2015 (20)	No

3’UTR, 3 prime untranslated region; 5’URR, 5 prime upstream regulatory region.

**Table 2:** Characteristics of studies included in the meta-analysis that reported the associations between HLA-G polymorphisms or sHLA-G expressions with RIF.

Study(year)	Period	Country	Race	N Case/Ctr	Age of Case/Ctr	Sample	Item analyzed	HWE(P-value)	Definition of RIF	Definition of control
Hviid (2004) (39)	NR	Denmark	Caucasian	14/108	NR	Blood	HLA-G 14-bp ins/del	0.042	Patients with ≥3 or more unsuccessful IVF treatments	Women with twin pregnancies after the first IVF treatment and fertile women with ≥2 uncomplicated pregnancies and births.
Sipak-Szmigiel (2007) (70)	NR	Poland	Caucasian	20/20	NR	Blood	HLA-G concentration	–	Patients with ≥3 or more unsuccessful IVF treatments.	Fertile women with ≥2 uncomplicated pregnancies and births.
Sipak Szmigiel (2009) (42)	NR	Poland	Caucasian	50/71	NR	Blood	HLA-G 14-bp ins/del HLA-G -725 C>G(T) HLA-G allele distribution	0.449 0.520 -	Patients with ≥3 or more unsuccessful IVF treatments.	Fertile women with ≥2 uncomplicated pregnancies and births.
Nardi (2012) (19)	NR	Brazil	Mixed	41/60	31.6 ± 5.6/44.2 ± 11.7	Blood	HLA-G concentration HLA-G allele distribution	–	Patients with ≥2 or more unsuccessful IVF treatments.	Fertile women with ≥2 normal pregnancies.
Enghelabifar (2014) (37)	NR	Iran	Caucasian	40/40	NR	Blood	HLA-G 14-bp ins/del	0.752	Women with at least two failed IVF-embryo transfers, using at least 6 appropriate cleaved embryos.	Women with pregnancy following IVF
Lashley (2014) (50)	2005-2009	Netherlands	Caucasian	24/96	35 (26–40)/33 (25–38)	Blood	HLA-G 14-bp ins/del	0.667	Patients with ≥3 or more unsuccessful IVF treatments with high quality embryos transferred.	Women with live birth after one IVF/ICSI treatment and fertile women with ≥1 uncomplicated pregnancies and births.
Kuroshli (2015) (20)	NR	Iran	Caucasian	100/50	32.3/NR	Blood	HLA-G alleles distribution	–	Patients with ≥2 unsuccessful IVF treatments	Healthy unrelated Iranian individuals
Nardi (2016) (35)	NR	Brazil	Mixed	49/34	36.04 ± 0.5/36.1 ± 1	Blood	HLA-G 14-bp ins/del HLA-G concentration	0.471 -	Patients with ≥2 or more unsuccessful IVF treatments.	Fertile women with ≥2 pregnancies.
Nowak (2020) (8)	NR	Poland	Caucasian	239/437	34.63 ± 4/31.97 ± 3.63	Blood	HLA-G 14-bp ins/del HLA-G -725 C>G(T) HLA-G concentration	0.843 0.616 -	Patients with ≥3 or more unsuccessful IVF treatments with good quality embryos transferred.	Women with live birth after one IVF/ICSI treatment and fertile women with ≥1 uncomplicated pregnancies and births.

NR, not reported; HWE, Hardy-Weinberg equilibrium; RIF, recurrent implantation failure; PCR, polymerase chain reaction; hCG, human chorionic gonadotrophin; ctr, control.

Ten studies are included in the systematic review to evaluate the associations between HLA-G polymorphism with sHLA-G expressions in patients attending IVF treatment. Characteristics of the ten studies are shown in **Table 3**. Four of the ten studies that reported the same HLA-G polymorphism with sHLA-G expression were subjected to meta-analysis. In summary, the studies were performed in Denmark, Poland, the Netherlands, and Brazil. Most of the study’s ethnicity is Caucasian. Seven studies reported the association of parental HLA-G polymorphism with peripheral blood plasma or serum sHLA-G expressions. Three studies reported the association of paternal HLA-G polymorphisms with seminal plasma sHLA-G expressions (77–79). One study detected the sHLA-G expression in the endometrium by IH (41). The other studies detected the sHLA-G expression in body fluid by ELISA with the detection limit range from 0.6U/ml to 3.9U/ml. Most studies detect sHLA-G isoforms of HLA-G1 and HLA-G5 by ELISA, except for one study that detected the sHLA-G isoforms of HLA-G5 and HLA-G6 (41). The quality assessment of all 11 studies for meta-analysis is listed in **Table 4**. Ten of the studies were assessed as high-quality, and one study assessed 6 points was defined as low-quality.

**Table 3:** Summary of the studies that reported the association of HLA-G polymorphisms with sHLA-G expression in patients attending IVF treatment.

Study	Country	Ethnicity	Study population	Number of patients tested	Sample tested	Timing of sample collection	Type of assay	sHLA-G isoform	Detection limit	Result	Included in meta-analysis
Papúchová 2022 (41)	Denmark	Caucasian	Women attending IVF treatment	121	Endometrium	the day equivalent to embryo transfer	IH	HLA-G5 and HLA-G6	NR	No significant differences were observed in sHLA-G expression between the different HLA-G genotypes	No
Piekarska 2021 (76)	Poland	Caucasian	Men attending IVF treatment	183	Seminal plasma	2-7 days of sexual abstinence	ELISA	HLA-G1 and HLA-G5	0.6 IU/ml	Certain HLA-G haplotypes and diplotypes of rs1632947-rs1233334-rs371194629 are associated with seminal sHLA-G expression	No
Nilsson 2020 (79)	Denmark	Caucasian	Couples attending IVF treatment	127 couples and 4 single women	Periphery blood plasma and seminal plasma	NR	ELISA	HLA-G1 and HLA-G5	0.6 IU/ml	HLA-G 14bp genotype or HLA-G 3’UTR diplotype is significantly associated with seminal sHLA-G levels, while is not significantly associated with female blood plasma sHLA-G levels	Yes
Nowak 2020 (8)	Poland	Caucasian	Women attending IVF	234 before IVF-ET and 185 after IVF-ET	Periphery blood plasma	Before and after IVF-ET	ELISA	HLA-G1 and HLA-G5	0.6 IU/ml	Certain HLA-G haplotypes of rs1632947-rs1233334-rs371194629 are associated with sHLA-G expression in blood plasma before IVF-ET; whereas such observations were not detected after ET	No
Craenmehr 2019 (77)	Netherlands	Caucasian	Men attending IVF treatment	156	Seminal plasma	NR	ELISA	HLA-G1 and HLA-G5	0.6 IU/ml	Seminal plasma sHLA-G levels are associated with HLA-G SNPs (HLA-G 14 bp ins/del, +3003 C/T, +3010 C/G, +3142 C/G, +3187 A/G, +3196 C/G, +3496 A/G and + 3509 G/T) and haplotypes at the 3’UTR	Yes
Nardi 2016 (35)	Brazil	Mixed	RIF and control patients	58	Periphery blood serum	NR	ELISA	HLA-G1 and HLA-G5	0.25ng/ml	HLA-G 14bp del variant is associated with higher sHLA-G levels	Yes
Dahl 2014 (78)	Denmark	Caucasian	Couples attending IVF treatment	43 men for seminal test; 53 women and 47 men for blood test	Seminal plasma and periphery blood plasma	NR	ELISA	HLA-G1 and HLA-G5	0.6 IU/ml	Male HLA-G 14bp del/del genotype is associated with higher sHLA-G levels in seminal plasma	Yes
Nardi 2012 (19)	Brazil	Mixed	RIF and control patients	41 RIF women and 60 controls	Periphery blood serum	NR	ELISA	HLA-G1 and HLA-G5	3.9U/ml	HLA-G allele distribution in exon 2,3,4 is not associated with serum sHLA-G levels	No
Sipak-Szmigiel 2007 (70)	Poland	Caucasian	Women attending IVF treatment	80	Periphery blood plasma	NR	ELISA	NR	2U/ml	Allele HLA-G10101, 10102, and 10108 was related to higher plasma sHLA-G levels than other alleles	No
Hviid 2004 (75)	Denmark	Caucasian	Couples attending IVF treatment	43 women and 42 male partners	Periphery blood serum	Before pregnancy	ELISA	HLA-G1 and HLA-G5	1ng/ml	Detectable sHLA-G in serum is restricted to HLA-G genotypes with the 14bp del	No

IH, Immunohistochemical staining; ELISA, enzyme-linked immunosorbent assay; IVF, *in vitro* fertilization; ET, embryo transfer; 3’UTR, 3’ untranslated region; 5’ URR, 5’ upstream regulatory region; NR, not reported.

**Table 4:** Newcastle-Ottawa Scale assessment of studies included in the meta-analysis.

Study (year)	Selection				Comparability		Exposure			Total Score	Quality
	Case definition adequate	Representativeness of the cases	Selection of controls	Definition of controls	Adjustment for age	Adjustment for other factors	Ascertainment of exposure	Uniform method of ascertainment	Non-response rate		
Hviid (2004)	*	*	*	*	–	–	*	*	*	7	High
Szmigiel (2007)	*	*	*	*	–	–	*	*	*	7	High
Szmigiel (2009)	*	*	*	*	–	*	*	*	*	8	High
Nardi (2012)	*	*	*	*	–	–	*	*	–	6	Low
Enghelabifar (2014)	*	*	*	*	–	–	*	*	*	7	High
Lashley (2014)	*	*	*	*	–	*	*	*	*	8	High
Dahl (2014)	*	*	*	*	–	–	*	*	*	7	High
Nardi (2016)	*	*	*	*	–	–	*	*	*	7	High
Craenmehr (2019)	*	*	*	*	–	–	*	*	*	7	High
Nowak (2020)	*	*	*	*	–	–	*	*	*	7	High
Nilsson (2020)	*	*	*	*	–	–	*	*	*	7	High

*denotes one score.

### Meta-analysis of association between HLA-G 14bp ins/del polymorphism and RIF

**Figure 2:**
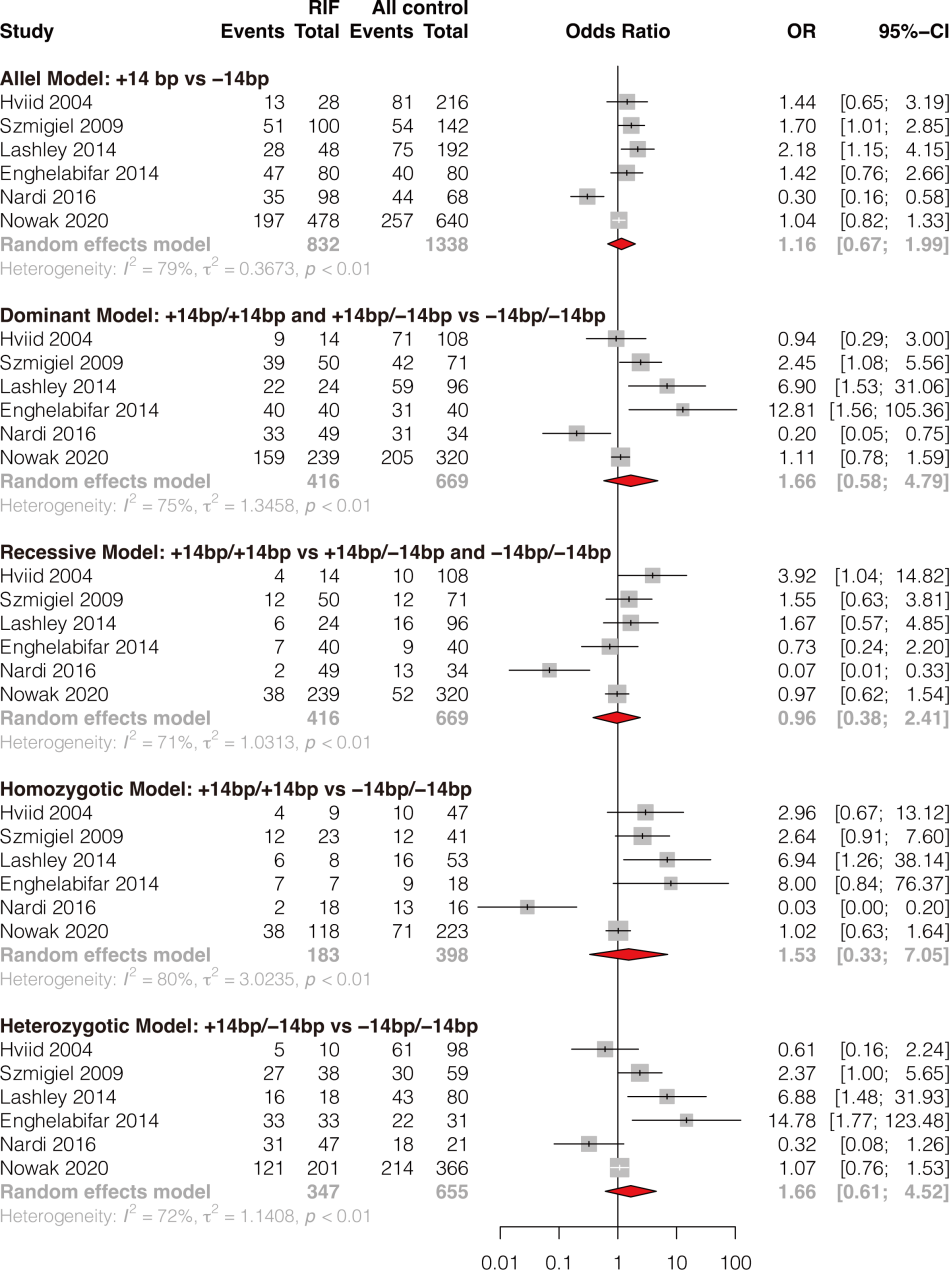
Forest plot showing the association of HLA-G 14bp ins/del polymorphism with RIF under 5 genetic models.

**Figure 3:**
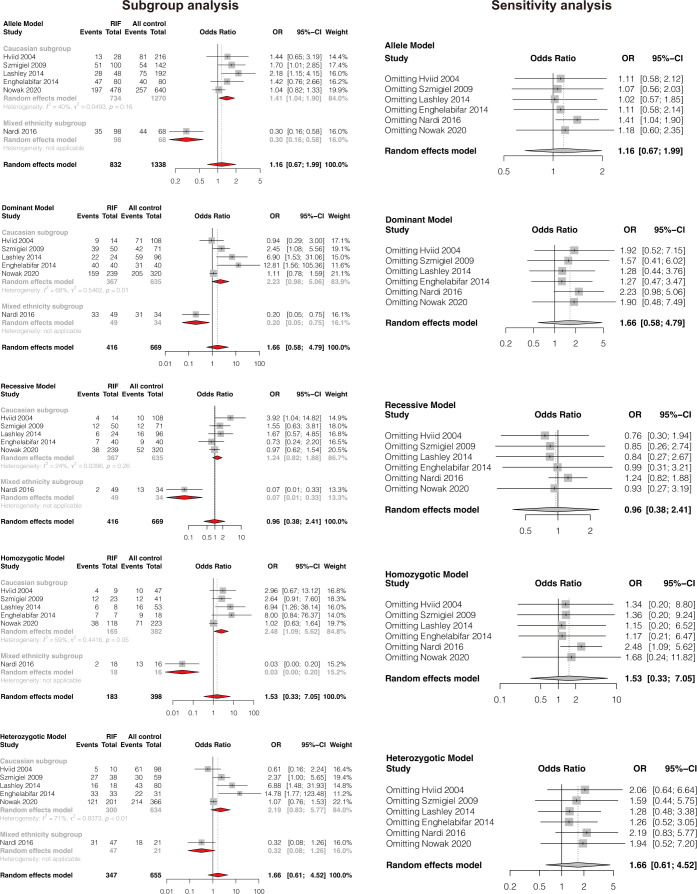
Forest plot showing subgroup analyses based on ethnicity and sensitivity analysis by omitting one study each time.

A total of six studies comprising 416 RIF cases and 669 controls were included (8, 35, 37, 39, 42, 50). Results showed that HLA-G 14bp ins/del polymorphism was not significantly associated with RIF in the general population under all genetic models (allele model: OR 1.16, 95%CI 0.67-1.99, p-value=0.599; dominant model: OR 1.66, 95%CI 0.58-4.79, p-value=0.344; recessive model: OR 0.96, 95%CI 0.38-2.41, p-value=0.925; homozygotic model: OR 1.53, 95%CI 0.33-7.05, p-value=0.584; heterozygotic model: OR 1.66, 95%CI 0.61-4.52, p-value=0.318) (**Figure 2** and **Supplementary Table 1**). Begg’s and Eggers’ tests showed no significant publication bias (**Supplementary Table 2**). Considering the effect of different genetic backgrounds on the results, we performed a subgroup meta-analysis based on different ethnicities. The subgroup analysis showed that HLA-G 14bp ins/del polymorphism was significantly associated with RIF in the Caucasian population under the allele model (OR 1.41, 95%CI 1.04-1.90, p-value=0.028) and the homozygotic model (OR 2.48, 95%CI 1.09-5.62, p-value=0.030) (**Figure 3** and **Supplementary Table 3**). Sensitivity analysis was performed by excluding one study each time; results of the sensitivity analysis showed that the result changed from non-significant to significant under the allele model (OR 1.41, 95%CI 1.04-1.90, p-value=0.028) and the homozygotic model (OR 2.48, 95%CI 1.09-5.62, p-value=0.030) after excluding Nardi’s study conducted in 2016 (**Figure 3**). It is worth noting that the study population in Nardi’s study conducted in 2016 was mixed ethnicity, while the population included in the other studies is Caucasian ethnicity. The subgroup analysis and sensitivity analysis consistently indicate that ethnic background plays an essential role in genetic susceptibility to RIF.

### Meta-analysis of association between HLA-G -725 C>G(T) polymorphism and RIF

**Figure 4:**
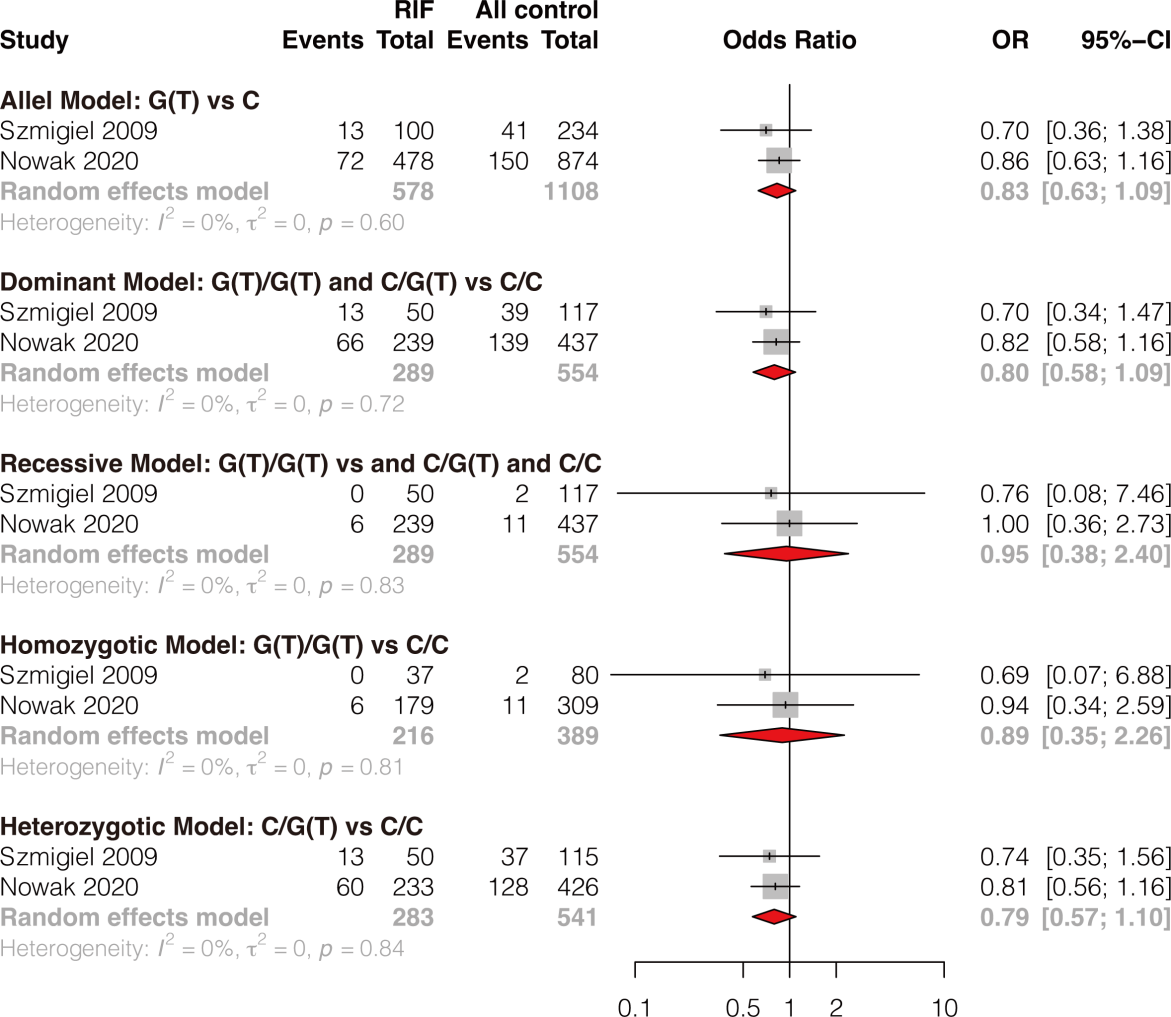
Forest plot showing the association of HLA-G -725C>G/T polymorphism with RIF under 5 genetic models.

Two studies reported the association of HLA-G -725C>G(T) polymorphism and RIF (8, 42). The results showed that single HLA-G -725C>G(T) polymorphism was not significantly associated with RIF under all genetic models (allele model: OR 0.83, 95%CI 0.63-1.09, p-value=0.183; dominant model: OR 0.80, 95%CI 0.58-1.09, p-value=0.155; recessive model: OR 0.95, 95%CI 0.38-2.40, p-value=0.920; homozygotic model: OR 0.89, 95%CI 0.35-2.26, p-value=0.813; heterozygotic model: OR 0.79, 95%CI 0.57-1.10, p-value=0.163) (**Figure 4** and **Supplementary Table 4**). Publication bias and sensitivity analysis were not performed due to the small number of included studies. Therefore, the results should be explained with caution because only two studies evaluated HLA-G -725 C>G(T) polymorphism and RIF, and the sample size is small.

### Meta-analysis of association between HLA-G alleles variants and RIF

**Figure 5:**
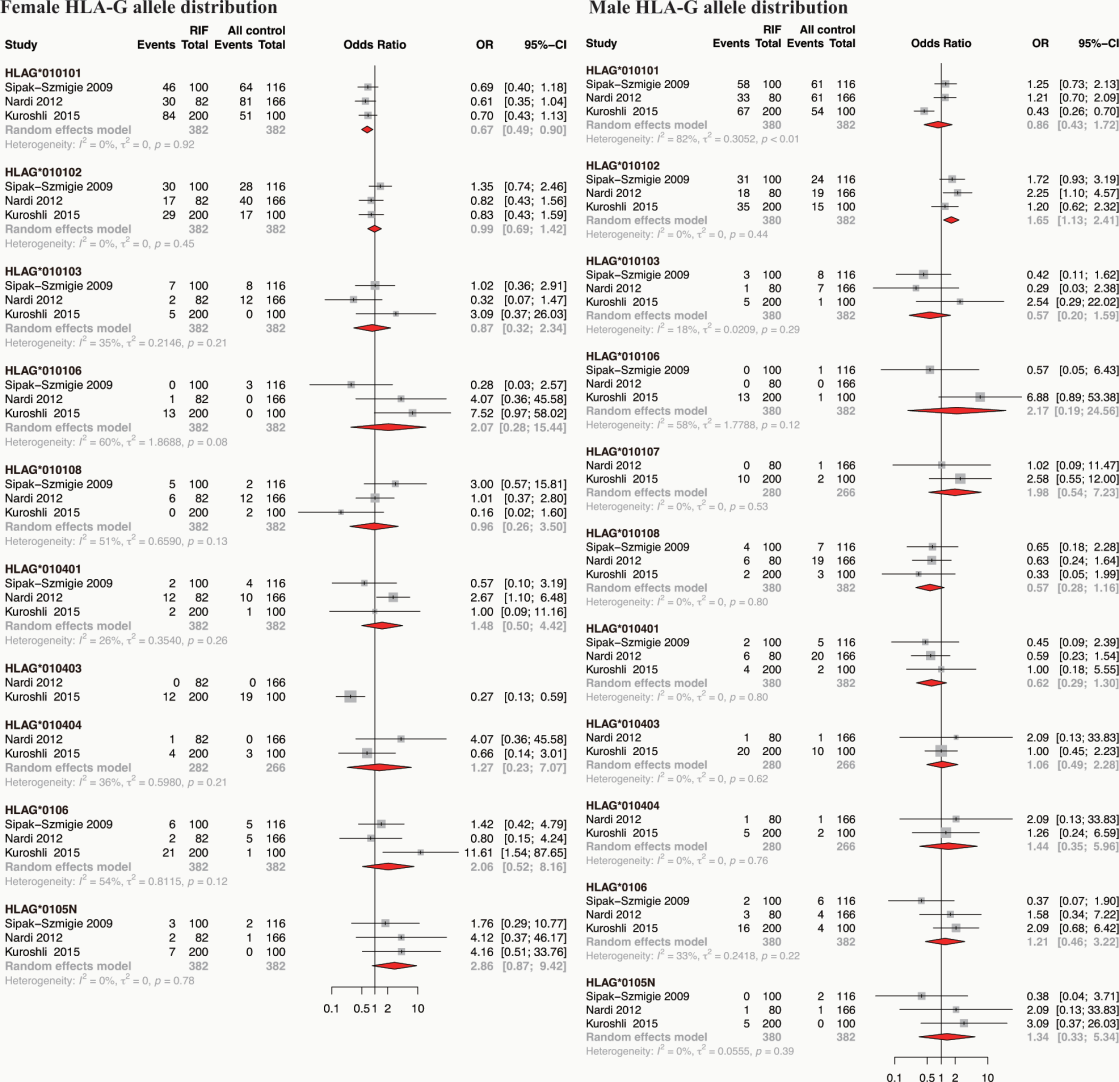
Forest plot showing the association of parental HLA-G allele distribution with RIF.

Three studies reported the association of HLA-G alleles distribution at exon 2-4 (HLA-G*010101, HLA-G*010102, HLA-G*010103, HLA-G*010106, HLA-G*010107, HLA-G*010108, HLA-G*010401, HLA-G*010403, HLA-G*010404, HLA-G*0106, and HLA-G*0105N) with RIF were included for meta-analysis. Results showed that the maternal HLA-G*010101 allele is associated with a lower risk of RIF (OR 0.67, 95%CI 0.49-0.90, p-value=0.008), maternal HLA-G*0105N tends to increase the risk of RIF though without statistical significance (OR 2.86, 95%CI 0.87-9.42, p-value=0.083). The paternal HLA-G*010102 allele is associated with a higher risk of RIF in their female partner (OR 1.65, 95%CI 1.13-2.41, p-value=0.010) (**Figure 5** and **Supplementary Table 5**).

### Meta-analysis of association between maternal circulating sHLA-G concentration and RIF

**Figure 6:**
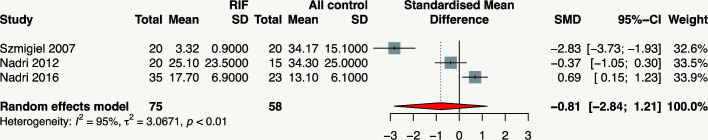
Forest plot showing the association of circulating sHLA-G concentration with RIF.

Three studies investigated the association of maternal circulating sHLA-G concentration with RIF (19, 35, 70). Meta-analysis showed that maternal circulating sHLA-G concentration was not significantly associated with RIF (SMD -0.81, 95%CI -2.84~-1.21, p-value=0.432) with extremely significant heterogeneity (I^2^= 95.4%, p-value<0.01) (**Figure 6** and **Supplementary Table 6**). The results should be explained cautiously due to the considerable heterogeneity and the small number of included studies.

### Meta-analysis of association between HLA-G polymorphism and sHLA-G expression in patients attending IVF treatment

**Figure 7:**
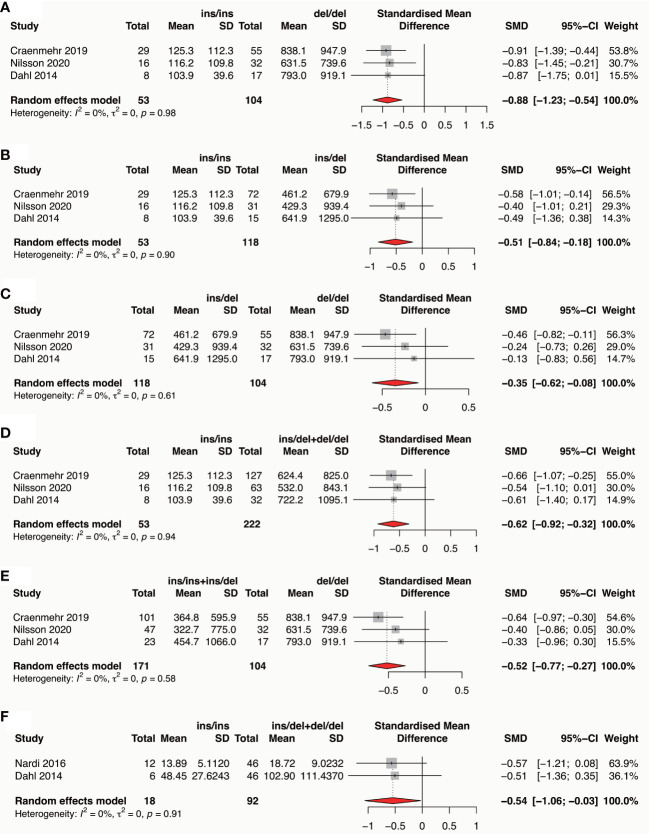
Forest plot showing the association of parental HLA-G 14bp ins/del polymorphism with sHLA-G expression in seminal plasma or in blood plasma. **(A)** Meta-analysis of sHLA-G expression in male seminal plasma in comparison of ins/ins genotype versus del/del genotype. **(B)** Meta-analysis of sHLA-G expression in male seminal plasma in comparison of ins/ins genotype versus ins/del genotype. **(C)** Meta-analysis of sHLA-G expression in male seminal plasma in comparison of ins/del genotype versus del/del genotype. **(D)** Meta-analysis of sHLA-G expression in male seminal plasma in comparison of ins/ins genotype versus ins/del+del/del genotype. **(E)** Meta-analysis of sHLA-G expression in male seminal plasma in comparison of ins/ins+ins/del genotype versus de/del genotype. **(F)** Meta-analysis of sHLA-G expression in female blood plasma in comparison of ins/ins genotype versus ins/del+del/del genotype.

Three studies reported the association of paternal HLA-G polymorphisms with seminal plasma sHLA-G expression (77–79), and two studies reported the association of maternal HLA-G polymorphisms with blood plasma sHLA-G expression (35, 78) were included for meta-analysis, respectively. Results showed that the paternal HLA-G 14bp ins variant is associated with a lower seminal sHLA-G expression in all comparisons (ins/ins vs del/del: SMD -0.88, 95%CI -1.23~-0.54, p-value<0.0001; ins/ins vs ins/del: SMD -0.51, 95%CI -0.84~-0.18, p-value=0.002; ins/del vs del/del: SMD -0.35, 95%CI -0.62~-0.08, p-value=0.010; ins/ins vs ins/del+del/del: SMD -0.62, 95%CI -0.92~-0.32, p-value<0.0001; ins/ins+ins/del vs del/del: SMD -0.52, 95%CI -0.77~-0.27, p-value<0.0001) (**Figure 7A-E** and **Supplementary Table 7**). Maternal HLA-G 14bp ins/ins genotype is associated with a lower blood plasma sHLA-G level than ins/del and del/del genotypes (SMD -0.54, 95%CI -1.06~-0.03, p-value=0.038) (**Figure 7F** and **Supplementary Table 8**). Due to incomplete data, we cannot conduct a meta-analysis based on other maternal 14bp genotype comparisons.

## Discussion

### Summary of this study

RIF is a multi-factorial complication following embryo transfer. In addition to the embryo factor, immune dysfunction is one of the leading factors contributing to implantation failure, which has been a focus of interest. HLA-G plays a central role in inducing immune tolerance through interactions between HLA-G and its receptors, including CD8, ILT-2/LILRB1/CD85j, ILT-4/LILRB2/CD85d, KIR2DL4, and NKG2A/CD94 (58, 80). Besides inducing immune tolerance, HLA-G controls trophoblast invasion, regulates vascular remodeling, and facilitates fetal growth, allowing for successful embryo implantation and pregnancy maintenance (81–85). HLA-G is predominantly produced by the extravillous trophoblasts (EVTs). The embryo-secreted sHLA-G increases during embryo development, from a relative 35% in the cleavage stage to 100% in the morula or blastocyst stage (86). The sHLA-G level in the embryo culture medium is a promising predictor of pregnancy outcome (28–32). Besides, immune cells in the endometrium and maternal peripheral antigen-presenting cells (APC) can be a source of sHLA-G (87). Decidualization by progesterone and cAMP can increase HLA-G expression (88). Immunohistochemistry (IH) experiments validated that sHLA-G is located in the endometrial stroma and glandular epithelium of pre-implantation and peri-implantation endometrium. sHLA-G expression is correlated with CD56+ uNK cell abundance and associated with pregnancy outcomes (41, 63). Several studies reported that abnormal sHLA-G expression is associated with pregnancy complications such as preeclampsia, recurrent miscarriage (RM), and recurrent implantation failure (RIF), and may be further linked to HLA-G polymorphisms (89, 90). Some meta-analyses have confirmed the association between HLA-G polymorphisms with susceptibility to preeclampsia (91, 92) and recurrent miscarriage (RM) (93, 94). The current systematic review and meta-analysis focused on the implication of the genetic variants responsible for altered HLA-G expression in relation to RIF. Our study indicates that specific HLA-G alleles or HLA-G polymorphisms are associated with sHLA-G expression in couples attending IVF treatment. Parental HLA-G*010101 and HLA-G*010102 alleles distribution is associated with RIF risk. However, single maternal HLA-G 14bp ins/del polymorphism, HLA-G -725 C>G/T polymorphism, or circulating sHLA-G concentration is not significantly associated with RIF in the general population. Whereas the HLA-G 14bp insertion variant is associated with RIF under a homozygotic genetic model in the Caucasian population.

### HLA-G 14bp ins/del polymorphism with reproductive disorders

HLA-G 14bp ins/del is the most commonly studied polymorphism of HLA-G. Although maternal HLA-G 14bp ins/del polymorphism was not significantly associated with RIF in the general population, the sensitivity analysis and the subgroup analysis consistently suggested that HLA-G 14bp ins/del polymorphism was significantly associated with RIF in the Caucasian population under the allele and homozygotic models. The subgroup analyses should be explained cautiously due to the small number of studies in the subgroup. Consistent with our study, a meta-analysis in 2017 reported similar results that the HLA-G 14bp ins/del polymorphism is related to RIF in Caucasian patients (9). However, compared to Fan’s meta-analysis in 2017, which only assessed the association between HLA-G 14bp ins/del polymorphism with RIF, our meta-analysis adds the associations between other HLA-G polymorphisms (including HLA-G -725C>G/T, multiple allele distributions at HLA-G exon2~4, specific haplotypes and diplotypes of HLA-G) with RIF. It adds the association between HLA-G polymorphism with sHLA-G expression as well as the association between sHLA-G expression with RIF susceptibility. Besides, the sample size in this systematic review and meta-analysis is the largest so far. Further studies are needed to confirm whether an association exists between HLA-G 14bp ins/del polymorphism with RIF in patients of other ethnicities. In addition to RIF, maternal HLA-G 14bp ins/del polymorphism is also reported to be associated with preeclampsia (95), gestational diabetes mellitus (96), and recurrent miscarriage (97–99). Almeida et al. combined implantation failure, preeclampsia, recurrent miscarriage, and spontaneous miscarriage as reproductive disorders and performed a meta-analysis; they found that the HLA-G 14bp ins variant is associated with reproductive disorders (92). Moreover, the HLA-G 14bp ins/ins genotype is associated with insulin resistance (100), birth weight, and placental weight (101). Nevertheless, other studies have observed contrary results (102–104). The contradictory findings may be explained by differences in ethnic background, sample size, and genotyping methodology.

### HLA-G -725 polymorphism with reproductive disorders

HLA-G -725C>G/T polymorphism at the promoter region is reported to change the methylation profile of CpG dinucleotide resulting in a modification of HLA-G expression (17). HLA-G -725C>G/T polymorphism is reported to be associated with male fertility (76), endometriosis progression (105), and miscarriage (17). Our study found no significant association of HLA-G -725C>G/T polymorphism with RIF under all genetic models. Sipak et al. reported that HLA-G 725 C>G/T polymorphism is not associated with pregnancy complications, including antiphospholipid syndrome, preeclampsia, intrauterine growth restriction, and recurrent spontaneous abortion (106). In summary, an association of HLA-G -725C>G/T polymorphism with pregnancy-related complications or pregnancy outcomes could not be confirmed.

### Other HLA-G polymorphisms or HLA-G haplotypes or diplotypes with reproductive disorders

Other HLA-G polymorphisms include HLA-G -964G>A (rs1632947), HLA-G haplotype of rs1632947–rs1233334–rs371194629, diplotypes of rs1632947–rs1233334–rs371194629, and HLA-G alleles distribution at exon2-4 and intron2 are potentially associated with RIF or reproductive outcomes following IVF-ET. HLA-G polymorphisms such as -716 G/T rs2249863 (107) and 3142C/G rs1063320 (108) have been reported to play a role in spontaneous abortion but have not been reported in RIF. Our results showed that the maternal HLA-G*010101 allele is associated with a lower RIF risk than other HLA-G alleles, whereas the paternal HLA-G*010102 allele is associated with a higher RIF risk. HLA-G*010101 and HLA-G*010102 are the most prevalent alleles compared to other HLA-G alleles and are associated with sHLA-G expressions. Whereas Warner et al. reported that the HLA-G*01011 allele has a statistically significant association with an enhanced chance of reproductive success following IVF-ET in Caucasian women (109). The HLA-G*0105N null-allele contains a deletion in exon 3, which leads to a frameshift, and no functional full-length HLA-G1 and -G5 protein can be expressed (110). Our meta-analysis observed a tendency that the HLA-G*0105N allele increased the RIF risk, while without statistical significance, possible because the sample size is too small to reach significance. Nonetheless, HLA-G1 and HLA-G5 are reported to be not essential for fetal survival, indicating that other HLA-G isoforms or other HLA molecules may compensate for the lack of HLA-G1 and HLA-G5 in immune modulation.

### Soluble HLA-G expression with reproductive disorders

Soluble HLA-G can be secreted into circulating blood, amniotic fluid, umbilical cord blood, and semen; it can be detected in non-pregnant/pregnant women, men, embryo/fetus, and the maternal-fetal interface. It is well-studied that the embryo-secreted sHLA-G level in the culture medium is a promising predictor for embryo implantation (28–31). However, the role of maternal sHLA-G expression on embryo implantation was less studied. The sHLA-G expression is different before pregnancy or during pregnancy. The blood sHLA-G level is higher in pregnant women than in non-pregnant women, and the sHLA-G level is higher in the first trimester of pregnancy than in the second and third trimesters (60, 111–113). It is reported that lower serum sHLA-G levels at the pre-ovulatory stage increase the risk of early miscarriage (61). A reduced frequency of HLA-G expressing CD4+T and CD8+ T cells in the peripheral blood is associated with RM and RIF (69). However, results concerning whether the maternal sHLA-G expression is associated with RIF are conflicting. This meta-analysis showed that single maternal sHLA-G expression was not significantly associated with RIF, while the heterogeneity is extremely significant (I^2^= 98%, p-value<0.01). The considerable heterogeneity may be caused by: (1) Each clinic applied a different ELISA system for detecting sHLA-G with different detection sensitivity or limits (114); (2) The timing of measurement during preimplantation development could be critical. Because sHLA-G expression before or after ET is different (8), it dynamically changes over the pregnancy weeks. Therefore, the timing of sHLA-G measurement may significantly affect the result; (3) Maternal circulating sHLA-G concentration might not be correlated with sHLA-G levels at the fetal-maternal interface. Whether maternal circulating sHLA-G levels are associated with fetal circulating sHLA-G levels is conflicting (111, 115); (4) Other pathological conditions or diseases also affect sHLA-G levels. Crohn’s disease, Behçet’s disease, multiple sclerosis, or organ transplant are associated with sHLA-G expression (116–119); (5) IVF factors such as maternal age, cycle type (frozen cycles or fresh cycles), or exogenous hormone supplementation may change sHLA-G levels (8). It is reported that HLA-G expression was positively associated with progesterone supplementation but negatively with estradiol (120) and maternal age; (6) The detection of sHLA-G level in most cases is limited to sHLA-G1/HLA-G5 isotype measured by ELISA due to restriction in antibodies. Whereas the expression pattern of the other isoforms is rarely analyzed. Different HLA-G isoforms may interact differently with the receptors; for example, ILT-2 only binds β2m-associated HLA-G1/G5 isotypes, while ILT-4 preferably binds β2m-free isoforms. How these isoforms of the HLA-G protein differ in function is poorly understood. A recent study detected endometrium sHLA-G5 and sHLA-G6 isotypes by IH; they found that endometrial sHLA-G5 and sHLA-G6 levels are higher in RIF patients compared to controls (41). Besides, sHLA-G levels are reported to be associated with oocyte competence (121, 122), endometriosis progression (123), pregnancy-related conditions such as SGA neonates (124), GDM (125), advanced labor (126), preterm premature rupture of membranes (127), intrauterine growth retardation (IUGR) (112), and preeclampsia (113, 128–130). Moreover, maternal circulating sHLA-G levels in the second trimester were significantly lower in pregnant women with 18-trisomy fetuses (T18) and significantly higher in those with 21-trisomy fetuses (T21) compared to the normal controls (131), and it is inversely correlated with fetal microchimerism levels (132). Also, there are contrary results that the HLA-G expression is similar between samples of normal and abnormal karyotypes, and there is no association between the HLA-G polymorphisms and altered expression in reduced abortion and miscarriage groups (133). Schallmoser et al. reported no association of sHLA-G expression with female reproductive outcomes following IVF-ET (134). More evidence is needed to determine whether maternal circulating sHLA-G expression is a predictor for RIF and whether a combination of maternal-, paternal- and embryo-derived sHLA-G levels have more clinical significance than single detection.

### Association of HLA-G polymorphism with sHLA-G expression

Multiple HLA-G polymorphisms are reported to be associated with sHLA-G expression. HLA-G 14bp ins/del polymorphism is the most commonly reported to be related to sHLA-G levels. Most studies indicate a significant association between HLA-G 14bp ins variant and reduced sHLA-G levels in maternal circulating blood or paternal semen (35, 75, 77, 78, 96, 104, 135–139). HLA-G 14bp del/del genotype is also associated with higher HLA-G on the trophoblast membrane (101). However, there are different results; two studies report that the HLA-G 14bp ins variant is associated with higher circulating sHLA-G levels (140, 141). The contradictory observations may have the following explanations: the correlation of sHLA-G expression and HLA-G polymorphism is affected by genetic backgrounds, pregnancy state, and diverse pathophysiologies or diseases. The expression pattern of HLA-G is different under various complications. Therefore, in this study, we review the associations of HLA-G polymorphism with sHLA-G expression in patients attending IVF treatment. Our meta-analyses observed that the parental HLA-G 14bp ins variant is associated with lower sHLA-G expression in female blood and male semen. The result indicates that parental HLA-G polymorphism may affect sHLA-G expressions in body fluid. However, further studies must confirm whether sHLA-G levels in parental body fluid affect pregnancy outcomes.

HLA-G -725C>G/T polymorphism is reported to be associated with sHLA-G levels in an *in-vitro* study, which found that JEG-3 cells with HLA-G -725G allele produces higher levels of sHLA-G compared to HLA-G -725C/T allele (142). Specific HLA-G allele distribution at exon2-4 is also reported to be associated with sHLA-G levels; for example, the HLA-G10101 allele is reported to be associated with higher sHLA-G levels in circulating blood (59, 70), while the HLA-G*01013 allele, HLA-G*0105N allele, or 1597ΔC null allele is associated with lower sHLA-G levels (143, 144). Three other SNPs in the 3’UTR are associated with HLA-G mRNA stability and sHLA-G levels: +3142 (rs1063320) substituting a C to a G, +3187 (rs9380142) substituting an A to a G, and +3196 (rs1610696) substituting a C to a G (15, 57, 145). Moreover, some polymorphisms in the 3’UTR can act as targets for miRNAs and control HLA-G mRNA stability and expression levels (146). Unfortunately, we cannot perform a meta-analysis based on those HLA-G polymorphisms in patients attending IVF treatment because of insufficient studies. Whether there is a combined effect of multiple HLA-G polymorphisms on sHLA-G expressions needs further investigation.

### Interaction of HLA-G with other HLA Ia and Ib genes in relation to reproductive disorders

We speculate that single maternal HLA-G polymorphism or circulating sHLA-G concentration is not a single major cause of implantation failure. Whereas combined genetic effect would have been more potent than a single polymorphism analysis (147). The association of HLA-G with RIF is more likely to depend on the combined HLA-G genetic effect rather than single polymorphisms in the 3’-UTR, 5’URR, or coding regions. The 14bp ins, in combination with the +3187A/A and +3142G/G SNP, plays a significant role in HLA-G mRNA regulation in human endometrial stromal cells (148). One single maternal genetic polymorphism or circulating sHLA-G level could not become an adequate independent cause of RIF because: (1) The mechanisms regulating maternal-fetal tolerance are complex. There are many immune mechanisms and other compensatory processes of maternal-fetal tolerance; (2) More than two HLA-G genotypic effects (maternal, paternal, and embryo) may participate in immune regulation during embryo implantation. Therefore, a single maternal or paternal HLA-G genotype cannot wholly reflect the immune state at the maternal-fetal interface; (3) The influence of clinical variables such as maternal age, gestational age, embryo factors, other diseases, or pathophysiological conditions were not controlled in the previous studies and can cause bias; (4) One gene has a variety of polymorphisms. The interaction of different polymorphisms of the same gene, the interaction of various genetic polymorphisms, and their combined effects on gene expression and molecular functions are not entirely understood. Except for HLA-G 14bp polymorphism, the other HLA-G genotypes/haplotypes/diplotypes at the 3’UTR, 5’URR, and exon regions, HLA-C polymorphism, HLA-F polymorphism, or HLA molecule receptor polymorphism as KIR is also associated with RIF (41, 149–151). Certain HLA-G variations are in linkage disequilibrium with three HLA-F locus SNPs that influence reproduction (152). HLA-G expression and function are under the control of miRNA *via* the miRNA binding site at HLA-G genes (146). Other cytokines such as IF-10, IFN-γ, LIF, PIF, and Galectin-1 can induce the production of HLA-G (88, 153–155). Rizzo et al. found that endometrium and uterine flushing fluid with high LIF, HB-EGF, Glycodelin-A, MCP1, IP10, HLA-G, and HLA-E, but low MUC-1 expression presented a higher permittivity to embryo implantation by an endometrial 3D *in vitro* model (156). In summary, multiple genetic factors, non-coding molecules, and cytokines together generate a pro-tolerance milieu network to regulate embryo implantation. Considering all of these factors, it is doubtful that one single molecule (sHLA-G) or a small genetic region could be uniquely associated with reproductive outcomes. At least a single HLA-G gene mutation, while it may contribute, is not a significant independent cause of RIF. The present challenge is to find the correct combination of genetic factors that define the susceptibility of RIF. More studies involving a careful selection of strictly-defined RIF patients and control patients, good-quality embryo transfers, and other essential molecules involved in embryo implantation (such as VEGFA, PAI-1, and MTHFR), studies controlling confounding factors are needed to determine whether the presence of HLA-G polymorphism have a crucial role in an immune imbalance during embryo implantation. And to what extent this could affect maternal-fetal tolerance and be linked to RIF.

### Limitations

As far as we know, this is the first systematic review and meta-analysis to investigate the association of HLA-G polymorphisms and sHLA-G expression in relation to RIF. The following potential limitations should be considered: (1) The validity of the meta-analysis depends on the internal validity of the included studies. We could only use the information provided at the study level. We could not analyze the unmeasured or unreported factors of the patient, such as maternal age, underlying causes of infertility, varying infertility treatment, or exogenous hormone supplementation; (2) The generalizability of our result is another limitation. Patients included in this study were mainly Caucasian. ‘RIF’ is a broad term, including heterogeneous causes and diagnoses; (3) HLA-G expression is multifactorial and can be influenced by many other factors such as splice variants, DNA methylation, miRNA-mediated post-transcriptional regulation, etc., which were not explored in this study.

### Future expectations and conclusions

The immuno-genetics of infertility is complex and might depend on different genes involved in embryo implantation. A better understanding of HLA-G allele structure and how the genetic diversity at regulatory sites shared by different alleles and haplotypes could affect its expression might shed further light on the comprehension of immuno-genetics mechanisms acting at the feto-maternal interface. The primary source of sHLA-G at the maternal-fetal interface is the embryo-derived trophoblasts. In contrast, most studies assessing the role of HLA-G in pregnant diseases have considered only the maternal genotype and ignored the contribution of the fetus and paternal partner. Ideally, mother-father-fetus genotypes should be tested. The significant association between HLA-G 14bp ins/del polymorphism, HLA-G -725 C>G/T polymorphism, or circulating sHLA-G concentration with RIF could not be confirmed in the general population. However, if and to what extent the use of the multiple polymorphisms combined with the sHLA-G test from parental body fluid and the culture medium might increase accuracy in the RIF prediction remains to be elucidated. In the future, testing multiple genetic variances or biomarkers at a time by a high-throughput method combined with machine learning may screen the best predictive model for RIF. Animal studies have shown that recombinant sHLA-G or synthetic HLA-G may have a therapeutic effect on arthritis disease or prolong the acceptance of skin grafts (157, 158). Whether recombinant sHLA-G or synthetic HLA-G can be used to treat reproductive disorders needs far more studies.

In conclusion, our study indicates that specific HLA-G alleles or HLA-G polymorphisms are associated with sHLA-G expression in couples attending IVF treatment. Several HLA-G polymorphisms may be associated with RIF, considering different ethnic backgrounds. A combined genetic effect should be considered in future studies to confirm the association of HLA-G polymorphisms and sHLA-G expressions in relation to RIF. Our findings will hopefully stimulate further research to identify whether multiple HLA-G genetics combined with the sHLA-G test of parental body fluid and the culture medium is of clinical relevance to implantation success.

## Data availability statement

The original contributions presented in the study are included in the article/**Supplementary Material**. Further inquiries can be directed to the corresponding author. The extracted data and statistical R scripts are uploaded in our Github (https://github.com/minizenghong/HLA-G_sHLA-G_RIF).

## Author contributions

HZ designed the study, performed the statistical analyses, drafted and revised the manuscript, tables, and figures. LH and DH searched the studies, extracted the data, and performed the quality assessment. All authors approved the submission of the manuscript.

## Funding

The study is supported by funds from Guangdong Basic and Applied Basic Research Foundation (grant number: 2021A1515110601). The study is supported by China Postdoctoral Science Foundation (grant number: 2022M711521).

## Conflict of interest

The authors declare that the research was conducted in the absence of any commercial or financial relationships that could be construed as a potential conflict of interest.

## Publisher’s note

All claims expressed in this article are solely those of the authors and do not necessarily represent those of their affiliated organizations, or those of the publisher, the editors and the reviewers. Any product that may be evaluated in this article, or claim that may be made by its manufacturer, is not guaranteed or endorsed by the publisher.
