# Supplementary material for: Association of parental HLA-G polymorphisms with soluble HLA-G expressions and their roles on recurrent implantation failure: A systematic review and meta-analysis
Source: Front Immunol. 2022 Dec 1;13:988370. doi: 10.3389/fimmu.2022.988370 (PMC9751038; doi:10.3389/fimmu.2022.988370)
Supplement: Supplementary file 1 [file DataSheet_1.docx]

**Supplementary materials**

**Searching strategy**

**Date of searching deadline: 19 Sep, 2022**

1. **For searching studies that evaluate the association of HLA-G polymorphisms with RIF:**

- Searching strategies in Pubmed:

((((HLA-G*) OR (HLAG*)) OR (human leukocyte antigen G)) AND ((((((((polymorphism) OR (mutation)) OR (allele)) OR (genotype)) OR (genetic)) OR (variant)) OR (haplotype)) OR (diplotype))) AND ((((((implantation) OR (in vitro fertilization)) OR (IVF)) OR (ICSI)) OR (Intracytoplasmic sperm injection)) OR (embryo transfer))

Returned **163** records

- Searching strategies in Embase:

('hla g' OR hlag OR 'human leukocyte antigen g') AND (polymorphism OR mutation OR allele OR genotype OR genetic OR variant OR haplotype OR diplotype) AND (implantation OR 'in vitro fertilization' OR ivf OR icsi OR 'intracytoplasmic sperm injection' OR 'embryo transfer')

Returned **119** records

- Searching strategies in CNKI:

( ( ( (主题=中英文扩展(HLA-G) 或者 题名=中英文扩展(HLA-G) 或者 v_subject=HLA-G 或者 title=HLA-G) 或者 (主题=人类白细胞抗原G 或者 题名=人类白细胞抗原G 或者 v_subject=中英文扩展(人类白细胞抗原G) 或者 title=中英文扩展(人类白细胞抗原G)) ) 并且 ( (主题=基因多态 或者 题名=基因多态 或者 v_subject=中英文扩展(基因多态) 或者 title=中英文扩展(基因多态)) 或者 (主题=基因型 或者 题名=基因型 或者 v_subject=中英文扩展(基因型) 或者 title=中英文扩展(基因型)) ) ) 并且 ( (主题=反复种植失败 或者 题名=反复种植失败 或者 v_subject=中英文扩展(反复种植失败) 或者 title=中英文扩展(反复种植失败)) 或者 (主题=反复着床失败 或者 题名=反复着床失败 或者 v_subject=中英文扩展(反复着床失败) 或者 title=中英文扩展(反复着床失败)) ) ) (模糊匹配)

Returned **5** records

- Grey literature searching returned **0** records

1. **For searching studies that evaluate the association of sHLA-G expressions with RIF:**

- Searching strategies in Pubmed:

((((((HLA-G*) OR (HLAG*)) OR (human leukocyte antigen G)) OR (sHLA-G*)) OR (sHLAG*)) AND (((expression*) OR (level*)) OR (concentration*))) AND ((((((implantation) OR (IVF)) OR (in vitro fertilization)) OR (ICSI)) OR (Intracytoplasmic sperm injection)) OR (embryo transfer))

Returned 188 records

- Searching strategies in Embase:

(implantation OR 'in vitro fertilization' OR ivf OR icsi OR 'intracytoplasmic sperm injection' OR 'embryo transfer') AND ('hla g' OR hlag OR 'human leukocyte antigen g' OR 'shla g' OR shlag) AND (expression OR level OR concentration)

Returned 274 records

- Searching strategies in CNKI:

( ( (主题=中英文扩展(HLA-G) 或者 题名=中英文扩展(HLA-G) 或者 v_subject=HLA-G 或者 title=HLA-G) 或者 (主题=人类白细胞抗原G 或者 题名=人类白细胞抗原G 或者 v_subject=中英文扩展(人类白细胞抗原G) 或者 title=中英文扩展(人类白细胞抗原G)) ) 并且 ( (主题=反复种植失败 或者 题名=反复种植失败 或者 v_subject=中英文扩展(反复种植失败) 或者 title=中英文扩展(反复种植失败)) 或者 (主题=反复着床失败 或者 题名=反复着床失败 或者 v_subject=中英文扩展(反复着床失败) 或者 title=中英文扩展(反复着床失败)) ) ) (模糊匹配),专辑导航：全部; 数据库：文献 跨库检索

Returned 11 records

- Grey literature searching returned **0** records

1. **For searching studies that evaluate the association of HLA-G polymorphisms with sHLA-G expression in patients attending IVF treatment:**

- Searching strategies in Pubmed:

(((((((HLA-G*) OR (HLAG*)) OR (human leukocyte antigen G)) OR (sHLA-G*)) OR (sHLAG*)) AND (((expression*) OR (level*)) OR (concentration*))) AND ((((((implantation) OR (IVF)) OR (in vitro fertilization)) OR (ICSI)) OR (Intracytoplasmic sperm injection)) OR (embryo transfer))) AND ((((((((polymorphism) OR (mutation)) OR (allele)) OR (genotype)) OR (genetic)) OR (variant)) OR (haplotype)) OR (diplotype))

Returned 98 records

- Searching strategies in Embase:

(polymorphism OR mutation OR allele OR genotype OR genetic OR variant OR haplotype OR diplotype) AND (implantation OR 'in vitro fertilization' OR ivf OR icsi OR 'intracytoplasmic sperm injection' OR 'embryo transfer') AND ('hla g' OR hlag OR 'human leukocyte antigen g' OR 'shla g' OR shlag) AND (expression OR level OR concentration)

Returned 85 records

- Searching strategies in CNKI:

( ( ( ( ( (主题=中英文扩展(HLA-G) 或者 题名=中英文扩展(HLA-G) 或者 v_subject=HLA-G 或者 title=HLA-G) 或者 (主题=人类白细胞抗原G 或者 题名=人类白细胞抗原G 或者 v_subject=中英文扩展(人类白细胞抗原G) 或者 title=中英文扩展(人类白细胞抗原G)) ) 并且 ( (全文=表达) 或者 (全文=水平) ) ) 并且 ( (主题=基因多态 或者 题名=基因多态 或者 v_subject=中英文扩展(基因多态) 或者 title=中英文扩展(基因多态)) 或者 (主题=基因型 或者 题名=基因型 或者 v_subject=中英文扩展(基因型) 或者 title=中英文扩展(基因型)) ) ) 并且 ( (主题=中英文扩展(sHLA-G) 或者 题名=中英文扩展(sHLA-G) 或者 v_subject=sHLA-G 或者 title=sHLA-G) 或者 (主题=可溶性人类白细胞抗原G 或者 题名=可溶性人类白细胞抗原G 或者 v_subject=中英文扩展(可溶性人类白细胞抗原G) 或者 title=中英文扩展(可溶性人类白细胞抗原G)) ) ) 并且 ( (全文=辅助生殖) 或者 (全文=胚胎移植) ) ) (模糊匹配),专辑导航：全部; 数据库：文献 跨库检索

Returned **3** records

- Grey literature searching returned **0** records

**Other resources 2**

**Supplementary Table 1 Results of meta-analysis that analyze the association of HLA-G 14bp ins/del polymorphism with RIF in five genetic models.**

| Genetic model | n | I^2^ (p-value) | Statistical effect model | OR | 95%CI | P-value |
| --- | --- | --- | --- | --- | --- | --- |
| Allele model | 6 | 79% (<0.01) | Fixed effect model | 1.1072 | 0.9242; 1.3265 | 0.2691 |
|  |  |  | Random effects model | 1.1564 | 0.6732; 1.9864 | 0.5985 |
| Dominant model | 6 | 75% (<0.01) | Fixed effect model | 1.3280 | 1.0049; 1.7549 | 0.0461 |
|  |  |  | Random effects model | 1.6646 | 0.5788; 4.7868 | 0.3444 |
| Recessive model | 6 | 71% (<0.01) | Fixed effect model | 0.9386 | 0.6754; 1.3042 | 0.7056 |
|  |  |  | Random effects model | 0.9568 | 0.3793; 2.4132 | 0.9254 |
| Homozygotic model | 6 | 80% (<0.01) | Fixed effect model | 1.1877 | 0.8259; 1.7082 | 0.3534 |
|  |  |  | Random effects model | 1.5314 | 0.3328; 7.0481 | 0.5842 |
| Heterozygotic model | 6 | 72% (<0.01) | Fixed effect model | 1.3047 | 0.9816; 1.7343 | 0.0670 |
|  |  |  | Random effects model | 1.6639 | 0.6128; 4.5180 | 0.3177 |

Notes: n: number of studies included; OR: odds ratio; CI: confidence interval.

**Supplementary Table 2 Results of Begg’s and Egger’s test to detect publication bias concerning HLA-G 14bp** **ins/del polymorphism.**

| Genetic model | n | P-value of Begg’s test | P-value of Egger’s test |
| --- | --- | --- | --- |
| Allele model | 6 | 0.8510 | 0.8359 |
| Dominant model | 6 | 0.3476 | 0.5307 |
| Recessive model | 6 | 0.5730 | 0.8157 |
| Homozygotic model | 6 | 0.5730 | 0.7151 |
| Heterozygotic model | 6 | 0.1885 | 0.4536 |

**Supplementary Table 3 Results of subgroup analysis based on ethnicity (HLA-G 14bp ins/del polymorphism).**

| Genetic model | Subgroup | n | I^2^ (p-value) | Statistical effect model | OR | 95%CI | P-value |
| --- | --- | --- | --- | --- | --- | --- | --- |
| Allele model | Caucasian | 5 | 39.8% (0.16) | Fixed effect model | 1.2486 | 1.0326; 1.5097 | 0.0220 |
|  |  |  |  | Random effects model | 1.4055 | 1.0372; 1.9047 | 0.0281 |
|  | Mixed | 1 | - | Mantel-Haenszel method | 0.3030 | 0.1587; 0.5785 | 0.0003 |
| Dominant model | Caucasian | 5 | 67.7% (0.01) | Fixed effect model | 1.5082 | 1.1260; 2.0200 | 0.0059 |
|  |  |  |  | Random effects model | 2.2257 | 0.9791; 5.0594 | 0.0562 |
|  | Mixed | 1 | - | Mantel-Haenszel method | 0.1996 | 0.0529; 0.7524 | 0.0173 |
| Recessive model | Caucasian | 5 | 24.4% (0.26) | Fixed effect model | 1.1564 | 0.8173; 1.6360 | 0.4119 |
|  |  |  |  | Random effects model | 1.2415 | 0.8209; 1.8778 | 0.3054 |
|  | Mixed | 1 | - | Mantel-Haenszel method | 0.0687 | 0.0142; 0.3321 | 0.0009 |
| Homozygotic model | Caucasian | 5 | 58.5% (0.02) | Fixed effect model | 1.5339 | 1.0430; 2.2559 | 0.0297 |
|  |  |  |  | Random effects model | 2.4799 | 1.0944; 5.6193 | 0.0295 |
|  | Mixed | 1 | - | Mantel-Haenszel method | 0.0288 | 0.0042; 0.1993 | 0.0003 |
| Heterozygotic model | Caucasian | 5 | 71.2% (<0.01) | Fixed effect model | 1.4152 | 1.0536; 1.9010 | 0.0211 |
|  |  |  |  | Random effects model | 2.1876 | 0.8296; 5.7685 | 0.1136 |
|  | Mixed | 1 | - | Mantel-Haenszel method | 0.3229 | 0.0826; 1.2620 | 0.1041 |

Notes: n: number of studies included; OR: odds ratio; CI: confidence interval.

**Supplementary Table 4 Results of meta-analysis that analyze the HLA-G -725 C>G/T polymorphism with RIF in five genetic models.**

| Genetic model | n | I^2^ (p-value) | Statistical effect model | OR | 95%CI | P-value |
| --- | --- | --- | --- | --- | --- | --- |
| Allele model | 2 | 0.0% (0.60) | Fixed effect model | 0.8267 | 0.6258; 1.0921 | 0.1804 |
|  |  |  | Random effects model | 0.8276 | 0.6263; 1.0937 | 0.1834 |
| Dominant model | 2 | 0.0% (0.72) | Fixed effect model | 0.7954 | 0.5807; 1.0894 | 0.1537 |
|  |  |  | Random effects model | 0.7957 | 0.5809; 1.0900 | 0.1547 |
| Recessive model | 2 | 0.0% (0.83) | Fixed effect model | 0.9516 | 0.3792; 2.3883 | 0.9159 |
|  |  |  | Random effects model | 0.9538 | 0.3794; 2.3981 | 0.9199 |
| Homozygotic model | 2 | 0.0% (0.81) | Fixed effect model | 0.8916 | 0.3538; 2.2467 | 0.8077 |
|  |  |  | Random effects model | 0.8942 | 0.3541; 2.2577 | 0.8129 |
| Heterozygotic model | 2 | 0.0% (0.84) | Fixed effect model | 0.7942 | 0.5747; 1.0975 | 0.1627 |
|  |  |  | Random effects model | 0.7943 | 0.5748; 1.0978 | 0.1631 |

**Supplementary Table 5 Results of meta-analysis that analyze the alleles distributions with RIF.**

| HLA-G alleles | n | I^2^ (p-value) | Statistical effect model | OR | 95%CI | P-value |
| --- | --- | --- | --- | --- | --- | --- |
| Females |  |  |  |  |  |  |
| HLAG*010101 | 3 | 0.0% (0.92) | Fixed effect model | 0.6655 | 0.4935; 0.8976 | 0.0076 |
|  |  |  | Random effects model | 0.6658 | 0.4936; 0.8982 | 0.0077 |
| HLAG*010102 | 3 | 0.0% (0.45) | Fixed effect model | 0.9875 | 0.6865; 1.4205 | 0.9460 |
|  |  |  | Random effects model | 0.9876 | 0.6858; 1.4222 | 0.9465 |
| HLAG*010103 | 3 | 35% (0.21) | Fixed effect model | 0.8461 | 0.4088; 1.7513 | 0.6526 |
|  |  |  | Random effects model | 0.8691 | 0.3234; 2.3357 | 0.7809 |
| HLAG*010106 | 3 | 60% (0.08) | Fixed effect model | 2.3361 | 0.8579; 6.3612 | 0.0969 |
|  |  |  | Random effects model | 2.0727 | 0.2782; 15.4448 | 0.4769 |
| HLAG*010108 | 3 | 51% (0.13) | Fixed effect model | 1.0229 | 0.4777; 2.1905 | 0.9535 |
|  |  |  | Random effects model | 0.9621 | 0.2643; 3.5027 | 0.9533 |
| HLAG*010401 | 3 | 26% (0.26) | Fixed effect model | 1.7453 | 0.8391; 3.6299 | 0.1361 |
|  |  |  | Random effects model | 1.4814 | 0.4967; 4.4182 | 0.4809 |
| HLAG*010403 | 1 | - | Fixed effect model | 0.2721 | 0.1262; 0.5867 | 0.0009 |
|  |  |  | Random effects model | 0.2721 | 0.1262; 0.5867 | 0.0009 |
| HLAG*010404 | 2 | 36% (0.21) | Fixed effect model | 1.1459 | 0.3253; 4.0365 | 0.8322 |
|  |  |  | Random effects model | 1.2735 | 0.2295; 7.0668 | 0.7821 |
| HLAG*0106 | 3 | 54% (0.12) | Fixed effect model | 2.5793 | 1.1896; 5.5925 | 0.0164 |
|  |  |  | Random effects model | 2.0560 | 0.5181; 8.1596 | 0.3054 |
| HLAG*0105N | 3 | 0.0% (0.78) | Fixed effect model | 2.9974 | 0.9233; 9.7309 | 0.0677 |
|  |  |  | Random effects model | 2.8634 | 0.8704; 9.4204 | 0.0834 |
| Male partner |  |  |  |  |  |  |
| HLAG*010101 | 3 | 82% (<0.01) | Fixed effect model | 0.8245 | 0.6116; 1.1115 | 0.2054 |
|  |  |  | Random effects model | 0.8569 | 0.4277; 1.7167 | 0.6631 |
| HLAG*010102 | 3 | 0.0% (0.44) | Fixed effect model | 1.6361 | 1.1156; 2.3996 | 0.0117 |
|  |  |  | Random effects model | 1.6489 | 1.1271; 2.4122 | 0.0100 |
| HLAG*010103 | 3 | 18% (0.29) | Fixed effect model | 0.5849 | 0.2386; 1.4336 | 0.2410 |
|  |  |  | Random effects model | 0.5709 | 0.2049; 1.5905 | 0.2836 |
| HLAG*010106 | 2 | 58% (0.12) | Fixed effect model | 3.1250 | 0.8278; 11.7971 | 0.0927 |
|  |  |  | Random effects model | 2.1661 | 0.1911; 24.5580 | 0.5327 |
| HLAG*010107 | 2 | 0.0% (0.53) | Fixed effect model | 2.0529 | 0.5898; 7.1453 | 0.2583 |
|  |  |  | Random effects model | 1.9762 | 0.5402; 7.2298 | 0.3033 |
| HLAG*010108 | 3 | 0.0% (0.80) | Fixed effect model | 0.5784 | 0.2856; 1.1714 | 0.1284 |
|  |  |  | Random effects model | 0.5742 | 0.2843; 1.1597 | 0.1219 |
| HLAG*010401 | 3 | 0.0% (0.80) | Fixed effect model | 0.6147 | 0.2951; 1.2802 | 0.1935 |
|  |  |  | Random effects model | 0.6193 | 0.2939; 1.3050 | 0.2077 |
| HLAG*010403 | 2 | 0.0% (0.62) | Fixed effect model | 1.0553 | 0.4879; 2.2828 | 0.8912 |
|  |  |  | Random effects model | 1.0578 | 0.4902; 2.2823 | 0.8862 |
| HLAG*010404 | 2 | 0.0% (0.76) | Fixed effect model | 1.4213 | 0.3370; 5.9944 | 0.6321 |
|  |  |  | Random effects model | 1.4351 | 0.3454; 5.9634 | 0.6192 |
| HLAG*0106 | 3 | 33% (0.22) | Fixed effect model | 1.2624 | 0.6061; 2.6291 | 0.5336 |
|  |  |  | Random effects model | 1.2116 | 0.4557; 3.2218 | 0.7004 |
| HLAG*0105N | 3 | 0.0% (0.39) | Fixed effect model | 1.3601 | 0.4107; 4.5038 | 0.6147 |
|  |  |  | Random effects model | 1.3356 | 0.3341; 5.3388 | 0.6823 |

**Supplementary Table 6 Results of meta-analysis that analyze the associations between maternal circulating sHLA-G concentration with RIF.**

| Comparison | n | I^2^ (p-value) | Statistical effect model | SMD | 95%CI | P-value |
| --- | --- | --- | --- | --- | --- | --- |
| RIF vs control | 3 | 95.4% (<0.01) | Fixed effect model | -0.2886 | -0.6711; 0.0939 | 0.1392 |
|  |  |  | Random effects model | -0.8116 | -2.8363; 1.2131 | 0.4321 |

**Supplementary Table 7 Results of meta-analysis that analyze the associations between HLA-G 14bp ins/del with sHLA-G expression in male seminal plasma.**

| Comparison | n | I^2^ (p-value) | Statistical effect model | SMD | 95%CI | P-value |
| --- | --- | --- | --- | --- | --- | --- |
| Ins/ins vs del/del | 3 | 0.0% (0.98) | Fixed effect model | -0.8818 | -1.2278; -0.5358 | <0.0001 |
|  |  |  | Random effects model | -0.8818 | -1.2278; -0.5358 | <0.0001 |
| Ins/ins vs ins/del | 3 | 0.0% (0.90) | Fixed effect model | -0.5122 | -0.8417; -0.1826 | 0.0023 |
|  |  |  | Random effects model | -0.5122 | -0.8417; -0.1826 | 0.0023 |
| Ins/del vs del/del | 3 | 0.0% (0.61) | Fixed effect model | -0.3495 | -0.6164; -0.0826 | 0.0103 |
|  |  |  | Random effects model | -0.3495 | -0.6164; -0.0826 | 0.0103 |
| Ins/ins vs ins/del+del/del | 3 | 0.0% (0.94) | Fixed effect model | -0.6201 | -0.9245; -0.3158 | <0.0001 |
|  |  |  | Random effects model | -0.6201 | -0.9245; -0.3158 | <0.0001 |
| Ins/ins+ins/del vs del/del | 3 | 0.0% (0.58) | Fixed effect model | -0.5192 | -0.7676; -0.2709 | <0.0001 |
|  |  |  | Random effects model | -0.5192 | -0.7676; -0.2709 | <0.0001 |

**Supplementary Table 8 Results of meta-analysis that analyze the associations between HLA-G 14bp ins/del with sHLA-G expression in female blood.**

| Comparison | n | I^2^ (p-value) | Statistical effect model | SMD | 95%CI | P-value |
| --- | --- | --- | --- | --- | --- | --- |
| Ins/ins vs ins/del+del/del | 2 | 0.0% (0.91) | Fixed effect model | -0.5448 | -1.0597; -0.0299 | 0.0381 |
|  |  |  | Random effects model | -0.5448 | -1.0597; -0.0299 | 0.0381 |
